# Early warning of West Nile virus mosquito vector: climate and land use models successfully explain phenology and abundance of *Culex pipiens* mosquitoes in north-western Italy

**DOI:** 10.1186/1756-3305-7-269

**Published:** 2014-06-12

**Authors:** Roberto Rosà, Giovanni Marini, Luca Bolzoni, Markus Neteler, Markus Metz, Luca Delucchi, Elizabeth A Chadwick, Luca Balbo, Andrea Mosca, Mario Giacobini, Luigi Bertolotti, Annapaola Rizzoli

**Affiliations:** 1 Dipartimento di Biodiversità ed Ecologia Molecolare, Centro Ricerca e Innovazione, Fondazione Edmund Mach, San Michele all'Adige, TN, Italia; 2 Istituto Zooprofilattico Sperimentale della Lombardia e dell’Emilia Romagna, Parma, Italia; 3 Cardiff University, School of Biosciences, The Sir Martin Evans Building, Museum Avenue, CF10 3AX Cardiff, Wales; 4 Istituto per le Piante da Legno e l'Ambiente - IPLA S.p.a., Torino, Italia; 5 Dipartimento di Scienze Veterinarie, Università degli Studi di Torino, Torino, Italia

**Keywords:** *Culex pipiens*, Population dynamics, Epidemiology, Linear-mixed models, Remote sensing

## Abstract

**Background:**

West Nile Virus (WNV) is an emerging global health threat. Transmission risk is strongly related to the abundance of mosquito vectors, typically *Culex pipiens* in Europe. Early-warning predictors of mosquito population dynamics would therefore help guide entomological surveillance and thereby facilitate early warnings of transmission risk.

**Methods:**

We analysed an 11-year time series (2001 to 2011) of *Cx. pipiens* mosquito captures from the Piedmont region of north-western Italy to determine the principal drivers of mosquito population dynamics. Linear mixed models were implemented to examine the relationship between *Cx. pipiens* population dynamics and environmental predictors including temperature, precipitation, Normalized Difference Water Index (NDWI) and the proximity of mosquito traps to urban areas and rice fields.

**Results:**

Warm temperatures early in the year were associated with an earlier start to the mosquito season and increased season length, and later in the year, with decreased abundance*.* Early precipitation delayed the start and shortened the length of the mosquito season, but increased total abundance. Conversely, precipitation later in the year was associated with a longer season. Finally, higher NDWI early in the year was associated with an earlier start to the season and increased season length, but was not associated with abundance. Proximity to rice fields predicted higher total abundance when included in some models, but was not a significant predictor of phenology. Proximity to urban areas was not a significant predictor in any of our models. Predicted variations in start of the season and season length ranged from one to three weeks, across the measured range of variables. Predicted mosquito abundance was highly variable, with numbers in excess of 1000 per trap per year when late season temperatures were low (average 21°C) to only 150 when late season temperatures were high (average 30°C).

**Conclusions:**

Climate data collected early in the year, in conjunction with local land use, can be used to provide early warning of both the timing and magnitude of mosquito outbreaks. This potentially allows targeted mosquito control measures to be implemented, with implications for prevention and control of West Nile Virus and other mosquito borne diseases.

## Background

West Nile virus (WNV) is a flavivirus of emerging public health relevance in Europe [1]. In nature it is maintained in enzootic cycles between avian reservoir hosts and mosquitoes. Humans are dead-end hosts in which infection can induce symptoms from mild flu-like fever to severe neurological syndromes such as meningitis, encephalitis, and acute flaccid paralysis [2].

Prevention by vaccination has been possible for horses since 2003, but a human vaccine is not yet available [3]. Discovered originally in Uganda in 1937 [4], WNV is now found on every continent except Antarctica [5]. Several epidemics have been documented in European countries during the last 4 years [1], and this recent upsurge in outbreaks within endemic areas, as well as the spread of the virus throughout the New World since 1999, have led to increasing health concerns [6]. Effective prevention and control policies are dependent on both a clearer understanding of the risk factors associated with infection, and advance warning of likely outbreaks.

Adequate mosquito density is critical for effective WNV transmission, and has a strong correlation with the number of human cases [7,8]. However, implementing mosquito control measures in response to reports of human cases typically is ineffectual because most humans have been infected by this time and cases appear at the end of the mosquito season, when populations are already in decline [1,9]. Early warnings of mosquito outbreaks would provide a much needed prediction of spill-over risk [10-12], enabling more timely control measures to be implemented, especially within WNV circulation areas.

Mosquitoes belonging to the *Culex pipiens* complex are thought to be the most efficient vectors for spreading WNV among birds, and from birds to humans and other mammals in the United States [13,14] as well as in Europe [15]. They are also involved in the transmission of other human and animal pathogens such as Usutu virus [16], avian malaria and filarial worms [17].

*Cx. pipiens* mosquitoes lay their eggs in water, and larval stages are aquatic. Aquatic habitats are therefore a prerequisite for mosquito populations, and rainfall is important in creating and maintaining suitable larval habitats [18], thus strongly affecting the abundance of adult mosquitoes [19]. Temperature also strongly influences distribution, flight behaviour and dispersal, and abundance of mosquitoes [18]. Specifically, temperature impacts on several aspects of the *Cx. pipiens* life cycle including development rates [20,21], gonotrophic cycle length [22] and diapause duration [23] as well as the duration of the extrinsic incubation period of the virus [24]. Urban infrastructure often provides key habitats for *Cx. pipiens*, reflecting its affinity for stagnant water and urban areas where artificial containers of water are numerous [12,25]. Vegetation density is also important, due both to a positive correlation with abundance of preferred avian host species [26], and because trees and shrubs may offer resting habitats and sugar sources to adults [27]. Mosquito population density therefore reflects a complex interaction among climate, land use and vegetation coverage.

In order to develop robust statistical models to predict mosquito population dynamics, detailed data are needed describing the phenology and abundance of mosquito populations, and associated environmental data at a suitable spatial and temporal resolution to act as predictor variables. Both the spatial and temporal range and resolution will determine the accuracy and range over which resulting model predictions can be made. In the Piedmont area of northern Italy, an extensive mosquito trapping programme has been in place since 1997, run by the Municipality of Casale Monferrato until 2006, and then by the Istituto per le Piante da Legno e l’Ambiente (IPLA). The area is at risk from WNV, having suitable vector and reservoir host populations, and increasing numbers of human cases of WNV in adjacent areas [28-30].

Detailed environmental data are available at suitable spatial and temporal resolution across the area, thus providing an excellent system to test predictors of mosquito population dynamics. Similarities of climate and land use [31] allow model predictions to be cautiously applied across northern Italy, where WNV has been circulating since 2008 [30].

Previously, part of this dataset (years 2000 to 2006) was used to test associations between weekly mosquito abundance (various species) and a range of environmental data, including land use and weekly averaged climate, during the time period 10–17 days prior to measures of mosquito populations [32]. This approach tested for predictors that immediately preceded short term variation in weekly mosquito abundance.

Here we followed a different approach, aiming to determine early warning predictors of between year variation in mosquito population dynamics. We focussed on *Culex pipiens* and we extended the dataset for analysis until 2011. The objective was to identify the best early warning predictors of annual variation in *Cx. pipiens* abundance and phenology, with the ultimate goal to guide entomological surveillance and thereby facilitate monitoring of WNV transmission risk.

## Methods

**Figure 1:**
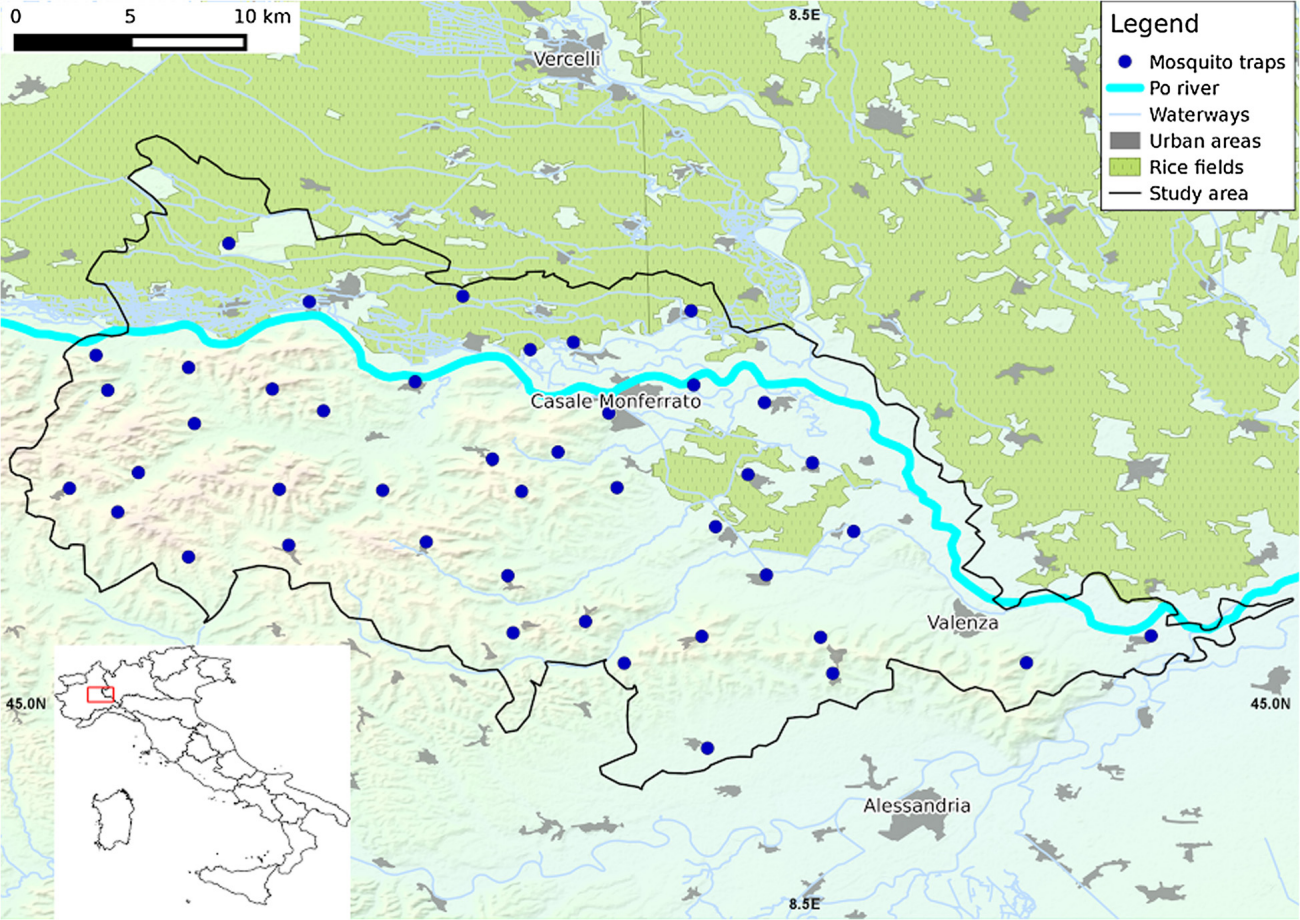
**Map of the study area.** Trap locations and land use are indicated. The map of Italy (inset) shows the location of the study area in the north west of the country.

The study area encompassed 987 km^2^ of the eastern Piedmont Region of north-western Italy (centroid: 45.07° N, 8.39° E) (Figure 1). There are highly suitable habitats for avian hosts of WNV, and breeding sites for mosquitoes, in close conjunction to human habitation. The landscape is primarily agricultural (mixed agriculture 72%, rice fields 14%), with areas of deciduous forest on the southern hills, and riverine habitat in the north (for further details see [32]). The climate is characterised by cold winters and warm summers (0.4 and 24°C respectively), and abundant precipitation (~600 mm/yr) primarily falling in the spring and autumn [32].

### Mosquito data

Mosquitoes were collected using CO_2_ baited traps, operated by Municipality of Casale Monferrato and the Istituto per le Piante da Legno e l’Ambiente (IPLA) [32]. Trapping sites were dispersed throughout the study area, with a minimum distance of 5 km between traps. Specific placement was based on coverage of all habitats deemed suitable for mosquitoes, in all participating municipalities, while enabling estimation of urban nuisance, and avoiding external disturbing factors (e.g. lighting, CO_2_ sources). Further details are provided in [32]. The current study includes data from 2001 to 2011, collected at 44 different sites (including 28–40 sites and an average of 37 sites activated each year) (Figure 1). Although most traps were run throughout, variation in activation at some sites occurred depending on the participation of individual municipalities in the scheme. Alongside monitoring efforts, mosquito control strategies have been implemented in the study area since 1998 [32]. However, the target of all treatments was *Ochlerotatus caspius*, and analyses (not presented here) showed that *Culex pipiens* mosquitoes were not affected by interventions.

Traps were set one night every week, for a twenty-week period starting at the beginning of May and ending in mid-September, thus encompassing the main period of mosquito activity. Traps were collected the following day, and the catch counted, sexed and identified. Each year since 2009, mosquitoes captured during a 6–7 night period at several sites (an average of 5 sites per year) have been pooled and tested for WNV. Until now no positive results have been found. For each trap, in every year, we (i) summed the total number of *Cx. pipiens* captured during the twenty-week survey period (TOTAL), (ii) calculated the week by which 5% and 95% of the population were captured, these being designated the start (ON) and end (OFF) of the mosquito season, respectively, and (iii) calculated the number of weeks between the arrival of 5% and 95% of the trapped population, this designated as season length (SEASL). As in [33], our definitions of ON and OFF are threshold values for population abundance, and do not necessarily reflect the cessation or initiation of diapause. Peak abundance within years was considered in preliminary analyses as a fourth measure of population dynamics, but was ill-defined and unpredictable, therefore results are not presented here.

### Environmental predictors

Environmental predictors were selected based on published evidence of their importance to mosquito populations [19,27,32,34].

All environmental data were processed in GRASS GIS [35], and extracted from the spatial database at the point corresponding with trap location. *Cx. pipiens* have a very limited dispersal (a few hundred metres [36]), which is within the pixel size for most spatial data (below), so data averaging over a wider area was not considered appropriate.

#### Climate

Precipitation was measured as total precipitation (TOT_PREC) and number of days of precipitation (DAY_PREC) from the gridded ECA&D (European Climate Assessment & Dataset, Version 8) [37,38] at approximately 25 km pixel resolution. Land surface temperature (LST) data were collated from the Moderate Resolution Imaging Spectroradiometer (MODIS) products MOD11A1 and MYD11A1, recorded twice daily. The original MODIS LST products were reconstructed at 250 m resolution, i.e. gap-filled to remove void pixels due to clouds [39,40]. For analyses, LST data were used to derive two values: (i) weekly mean LST, and (ii) a cumulative measure of temperature named here ‘growing degree weeks’ (GDW) (see [41]). This was derived by taking the positive difference in each week between mean LST and a threshold of 9°C (mosquitoes fail to develop below this threshold, see [20]). Weekly differences were summed cumulatively from the first week of the year, so that the *n*th GDW was obtained by summing the *n* consecutive differences (negative differences were assigned a value of zero).

#### Vegetation and water indices

Normalized Difference Vegetation Index (NDVI) was obtained from the MODIS product MOD13Q1, recorded every 16 days, and the Normalized Difference Water Index (NDWI) derived from the MODIS product MOD09A1, recorded every 8 days, both at 500 m resolution. For both the NDVI and the NDWI data, gaps were filled and outliers removed using a harmonic analysis of each time series [42]. These data were used as proxies for vegetation coverage (NDVI) [43] and for environmental water (NDWI), which includes surface water [44] as well as vegetation water content [43].

#### Land use

The distance from every sampling site to the nearest urban centre (DIST_URBAN) and rice field (DIST_RICE) was calculated using the Corine Land Cover raster dataset (using the CORINE classes 111 and 112 to map the urban settlements and 213 for the rice fields, [45], both at 100 m resolution).

### Temporal windows

We built 22 temporal windows by grouping periods of 12 consecutive weeks, starting from the first week of the year (weeks 1–12) and ending with weeks 22–33 (approximately the end of May to mid-August). The 22 windows were divided into two groups: the first ten windows (1–12, 2–13, etc., to 10–21) were designated the ‘early period’ and latter twelve windows (11–22, 12–23, etc., to 22–33) were designated the ‘late period’. The start of the mosquito season, ‘ON’, occurred on average during week 25, so our definition of early period predictors were those that were completed at least four weeks prior to this (i.e. ending weeks 10–21).

For each 12-week window, mean values were calculated for land surface temperature and vegetation indices (LST, NDVI and NDWI), whereas precipitation data were summed (TOT_PREC and DAY_PREC). For GDW, the cumulative value achieved by the end of the given window was used. Where these data are described in the text, the relevant temporal window is denoted in subscript, e.g. LST_1–12_ for mean land surface temperature during weeks 1–12.

The aggregation of 12 weeks was selected in order to test the effect of variations at a seasonal timescale and to avoid errors due to short term variation in mosquito collections. Comparisons with aggregation windows of alternative duration (1, 2, 4 and 8 weeks) proved this approach to be successful; twelve week windows produced more robust models and higher goodness-of-fit values, when compared to results obtained by aggregating data over shorter windows (see section A of Additional file 1 for details).

### Data analysis

We investigated the association between *Cx. pipiens* abundance (TOTAL) and seasonality (the start of the mosquito season, ON, and season length, SEASL, as defined above), and a range of environmental predictors. All statistical analyses were performed using R version 3.0.2 [46]. Dependent variables were transformed prior to analysis in order to normalize their distribution, following the Box-Cox method [47]. Transformations applied were *x*^1.3^ for ON and *x*^0.2^ for TOTAL while data for season length were normally distributed.

#### Preliminary analyses

Linear mixed effect models were used to ascertain, for each climatic variable, vegetation index and water index in turn, (i) which of the early period windows proved to be the best predictor of the start of the season (ON), and (ii) which of all the time windows (early and late) proved to be the best predictor of mosquito abundance (TOTAL) and season length (SEASL). In all models, trap identification number was included as a random variable. Models were ranked using the Akaike Information Criterion (AIC) [48], and for each climatic variable and vegetation/water index, the time window producing the lowest AIC was selected for inclusion in subsequent full models. For NDWI the first eight time windows were not included in preliminary analyses due to the potential presence of snow cover, which can dramatically alter the reliability of satellite acquisition of this parameter [49,50]. Terms that were not significant for any of the early or late time periods were not included in the full model. Variance Inflation Factor (VIF) [51] was used to test for collinearity between all explanatory variables. Where collinearity was significant (VIF values > 4, [51]), the variable producing the higher AIC was excluded. This led to the exclusion of GDW and total precipitation from further analyses. Vegetation and water indices were not correlated; however, NDVI was not significant in any of preliminary models, thus it was excluded from further analyses.

#### Full models

Following exclusion of collinear and non-significant variables, we developed linear mixed models including the remaining environmental variables, each measured over the optimum time window as selected through preliminary analyses. All two-way interaction terms were included in full models. In addition, we included distance to urban areas and to rice fields, and again included trap identification number as a random variable. Models were fitted in turn to predict (i) the start of the mosquito season (using early period predictors only), (ii) season length and (iii) mosquito abundance (modelled initially using only early period predictors, and then again using both early and late period predictors, in order to assess the additional variance explained by inclusion of the latter period).

Multi-model inference [52] was used to compare all possible models using the R package ‘MuMIn’ [53]. Models were ranked using AIC, and differences in AIC (ΔAIC) between consecutively ranked models were used to calculate weights and relative evidence ratios for each variable. The best models were selected using a threshold of ΔAIC ≤ 4 [52]. All variables included in the best models were ranked according to their importance (weight), i.e. the cumulative Akaike weight (wAIC) of the models that include that explanatory variable [53,54]. This provides an idea of the frequency with which the predictor was included in the most likely models, and not directly the importance of its effect on the predicted variable. Average coefficient for each variable was calculated following modelling average procedure [52].

In order to quantify the effect size of each predictor variable, predictions were made from the best models for each significant predictor variable in turn. For predictive models, all variables but one were fixed at their average values, and predictions made across the full range of the selected variable. For example, to test the association between temperature and the start of the mosquito season (ON), in a model where temperature, precipitation and NDWI were significant predictors, precipitation and NDWI were entered into the model as constants (fixed at their average measured value), while values for temperature were allowed to vary within their observed range. Models and plots were created using transformed data (for ON and TOTAL); predictions described in the text use back-transformed values to aid interpretability.

## Results

### Mosquito indices

**Figure 2:**
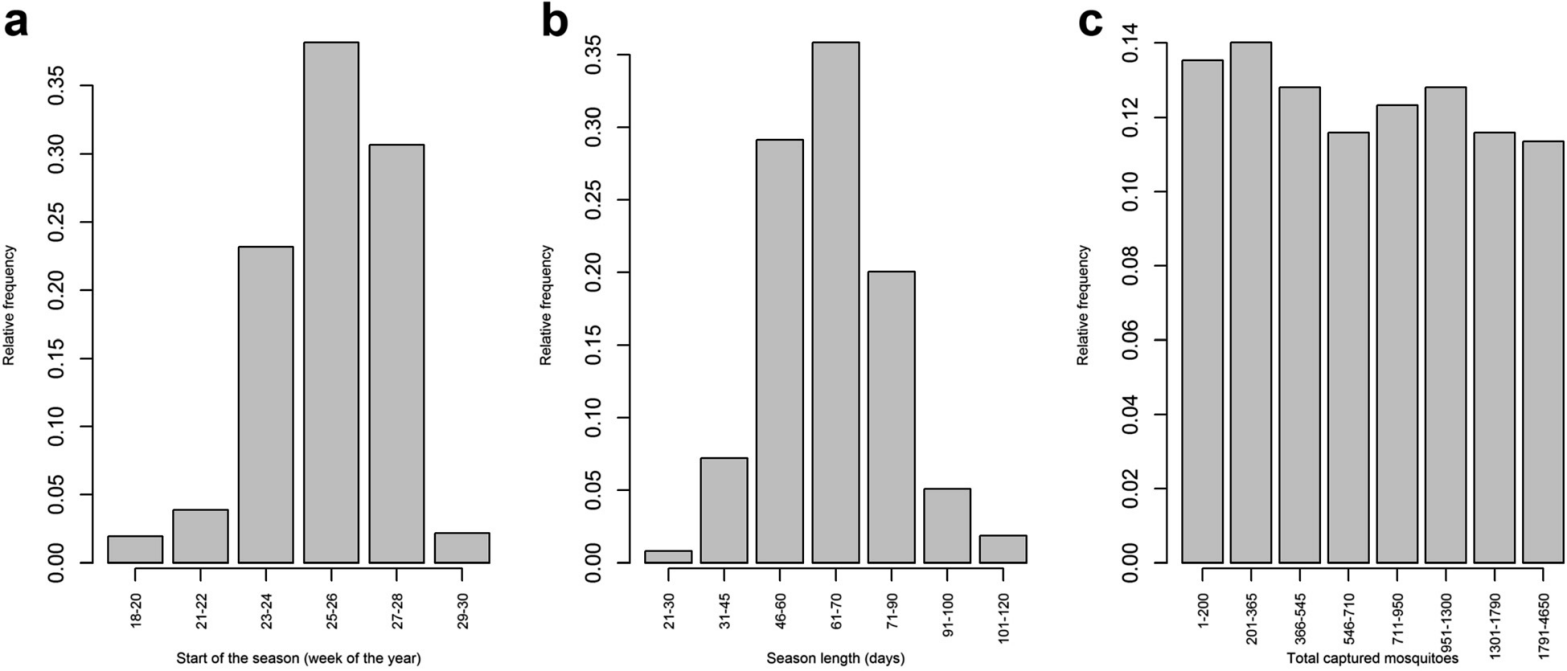
**Timing and abundance of the mosquito season.** Frequency distributions for **(a)** the start of mosquito season (the date by which 5% of total captures were made), **(b)** season length (the period in days between the collection of 5% and 95% of the captured population) and **(c)** the total number of *Cx. pipiens* captured.

The start of the mosquito season (ON) typically occurred during weeks 24–27 of the year (Figure 2a), and the main capture period (SEASL) lasted for 56–70 days (Figure 2b). The number of individuals captured (TOTAL) varied between 44 and 4648 per trap per year; more precisely, for one third of the traps the observed abundances varied between 44 and 500, for another third between 500 and 1000 and the remainder between 1000 and 4648 individuals (Figure 2c).

### Model results

#### Preliminary analyses

For prediction of the start of the season (ON), the optimum time windows selected for inclusion in the model were weeks 8–19, 6–17, and 10–21 for temperature (LST), precipitation (DAY_PREC) and NDWI respectively (determined by comparison of AICs, see Figure B1 in Additional file 1). For prediction of season length (SEASL) using only early period predictors, the optimum windows for temperature and NDWI were the same as for prediction of ON (8–19; 10–21) but the optimum window for precipitation was earlier, weeks 2–13. Late period predictors were weeks 16–27 for temperature, 20–31 for precipitation and 11–22 for NDWI (see Figure B1 in Additional file 1). For prediction of mosquito abundance (TOTAL) using only early period predictors, the optimum windows for temperature and precipitation were weeks 10–21 and 1–12, respectively; NDWI was not significant for any time window. Additional late period predictors were weeks 21–32, 15–26 and 22–33 for temperature, precipitation and NDWI respectively (see Figure B1 in Additional file 1).

#### Full models

**Figure 3:**
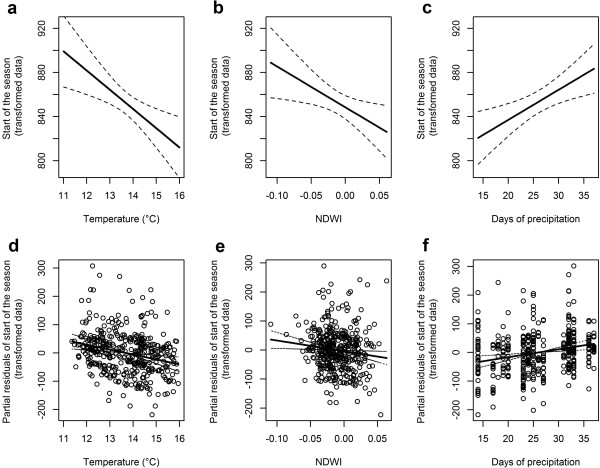
**Association between the start of the mosquito season and environmental variables.** Panels **a-c** show model predictions; panels **d-f** show partial residuals. The first column **(a,d)** shows the association between the start of the season and temperature (LST_8–19_), the second **(b,e)** shows the association with NDWI_10–21_ and the third **(c,f)** shows the association with precipitation (DAY_PREC_6–17_). Note that all plots show transformed data on the *y* axis (i.e. *x*^1.3^); back transformed values are presented in the text to assist interpretation.

For the start of the season (ON) 32 full models were produced and a single best model was selected, explaining 26% (R^2^ = 0.258, Akaike weight = 0.96) of the variance; remaining models had ΔAIC > 4 and were disregarded (see section C of Additional file 1 for more details). Model outputs (Table 1) are therefore based on a single model, rather than averages from multiple models as elsewhere. Within the measured range of environmental data, temperature had the greatest effect on the start of the season. Higher spring temperatures were associated with an earlier start to the season, such that an increase of 5°C in LST_8–19_ (from 11 to 16°C) predicts the start of the season some 14 days earlier (a shift in the average ON from day 187 to 173) (Figure 3a). Increasing NDWI also predicts an earlier start to the season, such that a shift in NDWI_10–21_ from -0.1 to +0.06 led to a start of the season 10 days earlier (Figure 3b), while more days of precipitation delayed the start of the season such that an increase in DAY_PREC_6–17_ from 14 to 37 days of precipitation during the 12 week period led to a delay in the start of the season of 10 days (Figure 3c). All terms selected in the best models (LST_8–19,_ NDWI_10–21_ and DAY_PREC_6–17_) were highly important with a predictor weight equal to or very close to 1 (Table 1). Neither distance to urban area or rice fields were significant predictors.

**Table 1:** Predicting the start of the mosquito season (ON)

**Variable**	**Weight**	**Coeff.**	**Std. error**	**z-value**	**Pr(>|z|)**
Intercept		1014.19	96.54	10.51	<0.001
LST_8–19_	1	-17.3	5.6	-3.09	0.002
NDWI_10–21_	1	-369.07	155.43	-2.37	0.018
DAY_PREC_6–17_	0.99	2.76	0.88	3.12	0.002

The weight and significance of terms remaining in the best selected model.

**Figure 4:**
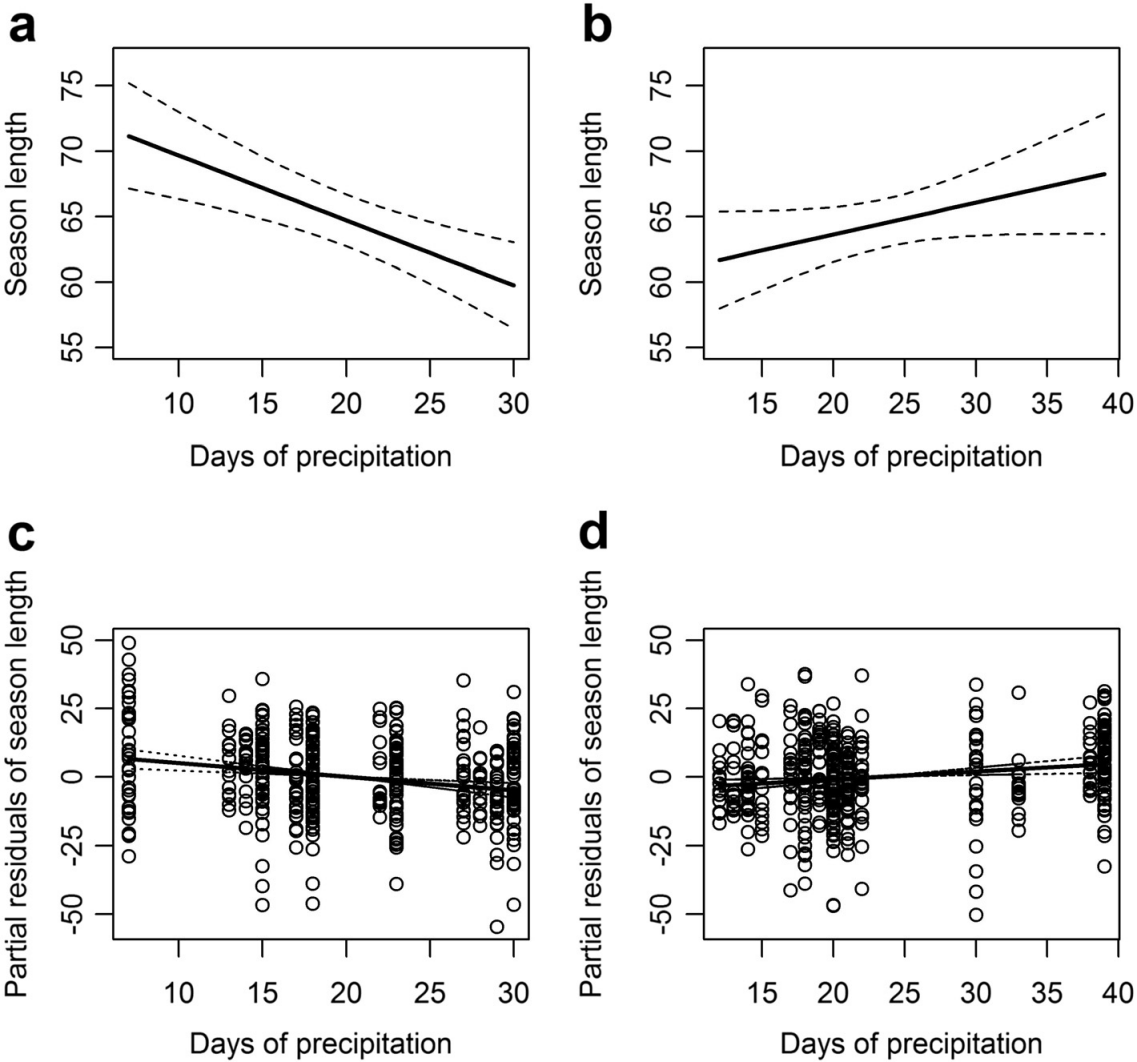
**Association between season length and days of precipitation.** Panels **a-b** show model predictions; panels **c-d** show partial residuals. The first column **(a,c)** shows the association with days of precipitation during the early period (DAY_PREC_2–13_) while the second column **(b,d)** shows the association with precipitation in the late period (DAY_PREC_20–31_).

When considering only the early period, two models, out of 32 models produced, were selected to predict season length, explaining between 13 and 14% (R^2^ = 0.135, R^2^ = 0.141) of the variance, and differed in their inclusion/exclusion of temperature (Akaike weights were 0.21 and 0.77). From model averaging, the early period variables associated with earlier start of the season (ON, above) also predict increased season length, so higher NDWI and temperature predict a longer season (although note that following averaging procedures temperature is significant only at a 92% threshold, with p = 0.079), and more days of precipitation predict a shorter season. Again, distance to urban areas and rice fields were not significant predictors, for either of the two best models. For early period predictors only, an increase in NDWI_10–21_ from -0.1 to +0.06 predicts an increase of 14 days in season length (from 56 to 70 days), while an increase in days of precipitation from 7 to 30 days during the 12 week period (DAY_PREC_2–13_) predicts an eleven day decrease in season length (from 71 to 60 days) (Figure 4a). An increase of 5°C in LST_8–19_ (from 11 to 16°C) predicts an extension of 7 days in season length (from 65 to 72 days).

**Figure 5:**
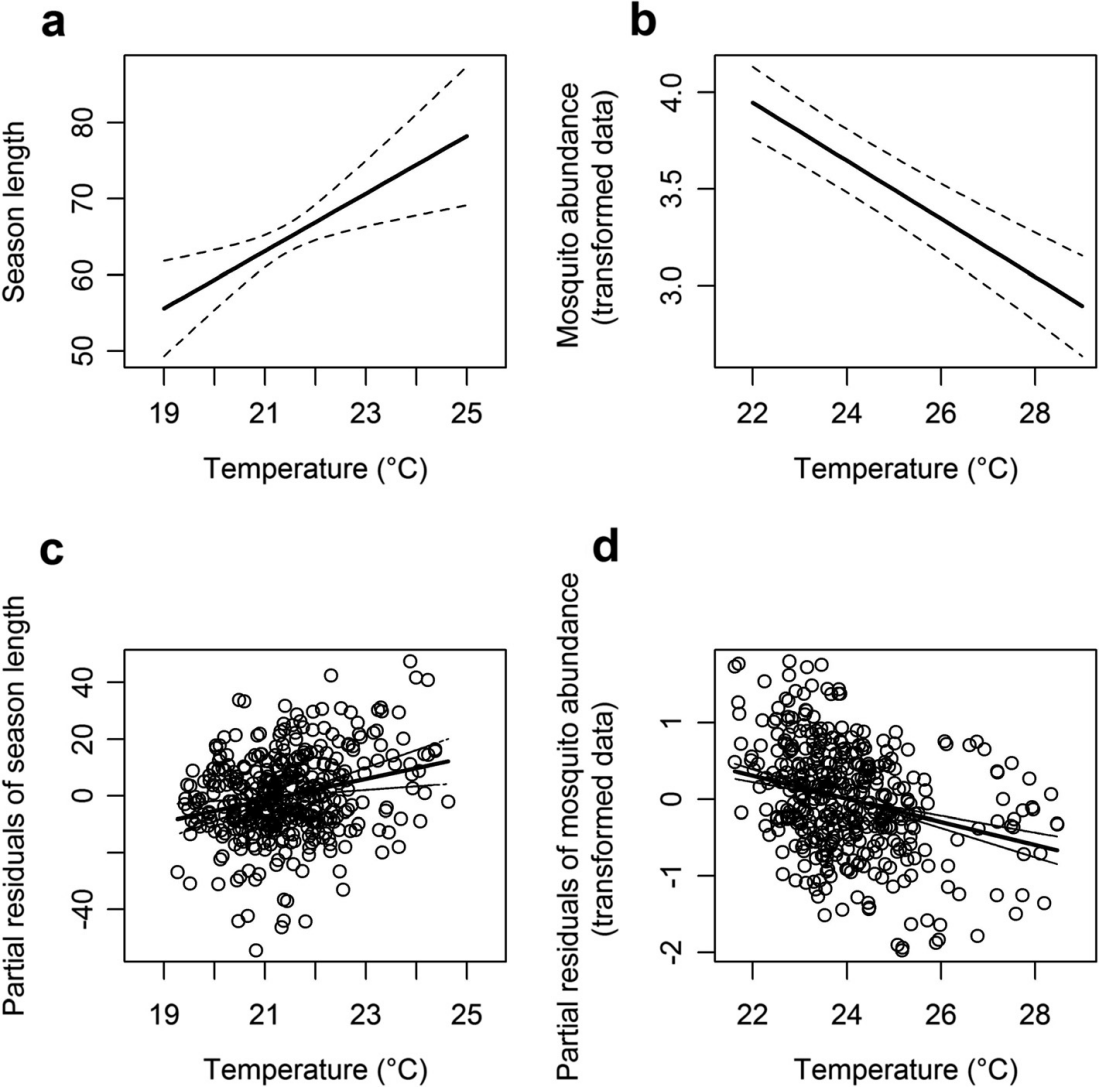
**Association between season length, total abundance and late season temperatures.** Panels **a-b** show model predictions; panels **c-d** show partial residuals. The first column **(a,c)** shows the association between late season temperature (LST_16–27_) and season length; the second column **(b,d)** shows the association between late season temperature (LST_21–32_) and mosquito abundance. Note that plots in the second column show transformed data on the *y* axis (i.e. *x*^0.2^); back transformed values are presented in the text to assist interpretation.

When incorporating late period variables in addition to early period, 128 full models were produced and six of them were selected as best, with R^2^ between 0.147 and 0.160 and Akaike weights between 0.28 and 0.06. Improvement to the model fit from inclusion of late period variables was therefore minimal, when compared to early period predictors alone (see above). Comparison of the model terms suggests, however, that precipitation during the late period (DAY_PREC_20–31_) has the opposite effect of precipitation during the early period (DAY_PREC_2–13_) (Figure 4b). More days of precipitation during the late period predict a longer season, such that an increase from 12 to 39 days of precipitation (DAY_PREC_20–31_) predicts a seven day increase in season length, whereas in the early period only model, more days of precipitation delay the season start and so shorten season length (as described above). The association with late period precipitation is stronger than that of early period precipitation, so that when both terms are included in the same model, early period precipitation becomes non-significant with a predictor weight of only 0.4, as compared to a high significance of p= 0.004 and a weight of 0.79 for late period precipitation (Table 2). Late period temperatures (LST_16–27_) have a marked impact on season length such that a shift of 6°C (from 19 to 25°C) predicts a lengthening of the season by 22 days (Figure 5a). As for precipitation, the addition of late period temperature renders early period temperature non-significant, with predictor weight of only 0.53, as compared to late period temperature which is both highly significant (p = 0.003) and has a high predictor weight (0.98) (Table 2). The most important model term in terms of predictor weight was, however, NDWI measured during the early period (NDWI_10–21_), which is positively associated with season length, and retains the same high predictor weight (1) in both groups of models (early only, early + late) (Table 2). An increase in NDWI_10–21_ from -0.1 to +0.06 predicts an increase in season length of 14 or 17 days (the greater increase being predicted by the early + late models).

**Table 2:** Predicting season length (SEASL)

**Model**	**Variable**	**Weight**	**Coeff.**	**Std. error**	**z-value**	**Pr(>|z|)**
**Early**	Intercept		59.57	15.42	3.86	< 0.001
	NDWI_10.21_	1	85.23	31.52	2.7	0.007
	DAY_PREC_2–13_	0.99	-0.5	0.14	3.65	< 0.001
	*LST*_*8–19*_	*0.78*	*1.5*	*0.85*	*1.76*	*0.079*
**Early + Late**	*Intercept*		*-19.11*	*28.45*	*0.67*	*0.501*
	NDWI_10–21_	1	104.26	31.36	3.32	0.001
	LST_16–27_	0.98	3.78	1.26	2.98	0.003
	DAY_PREC_20–31_	0.79	0.29	0.1	2.88	0.004
	*LST*_*8–19*_	*0.53*	*0.1*	*1.11*	*0.09*	*0.926*
	*DAY_PREC*_*2–13*_	*0.4*	*-0.28*	*0.16*	*1.73*	*0.083*

The average weight and significance of variables remaining in the two best 'Early predictors only' and six best 'Early + Late predictors' models. Note that terms in italics are significant in some of the selected best models but not in others, and that overall, weighted model averaging procedures suggest that they are not significant.

Of the 16 full models produced, two were selected to predict mosquito abundance (TOTAL) from early period predictors, explaining between 46 and 49% of the variance (R^2^ = 0.464, R^2^ = 0.488) with Akaike weights of 0.12 and 0.79 respectively. Abundance was best predicted by early period models including days of precipitation at the start of the year (DAY_PREC_1–12_), and distance to rice fields. An increase in precipitation predicts an increase in abundance (e.g. an increase from 7 to 30 days rain predicts an increase from approximately 400 to 1000 mosquitoes per trap). Traps closer to rice fields captured more mosquitoes than those 13 km away (average 680 mosquitoes per trap year, compared to 560). The very different prediction weights of the two terms selected in the early period models (Table 3), however, indicate that while days of precipitation play an important role, distance to rice fields has a very limited effect on early period model predictions. Incorporation of additional late period predictors did not greatly improve the model fit; again, two models were selected, out of 128 models produced, and explained 52% of the variance (R^2^ = 0.523, R^2^ = 0.524) with Akaike weights of 0.35 and 0.49 respectively. Days of precipitation at the start of the year (DAY_PREC_1–12_) remained a highly significant predictor, and predicted a similar effect (an increase from 7 to 30 days of rain predicts an increase in total abundance from 420 to 860 mosquitoes per trap year). Distance to rice fields was not a significant predictor in early + late period models, while average temperature during the late period (LST_21–32_) exerted a significant negative effect on predictions, such that an increase in temperature from 21 to 30°C led to a marked decrease in abundance from approximately 1150 to only 150 mosquitoes per trap year (Figure 5b). The days of precipitation measured during the early period (DAYPREC_1–12_) is the most important term predicting TOTAL in both groups of models (early only, early + late) while temperature has a strong impact on model prediction for the early + late model only (Table 3). Late period NDWI (NDWI_22–33_) was selected only in one of the best models and following model averaging was not significant.

**Table 3:** Predicting mosquito abundance (TOTAL)

**Model**	**Variable**	**Weight**	**Coeff.**	**Std. error**	**z-value**	**Pr(>|z|)**
**Early**	Intercept		1.27	8.4e-03	152.83	< 0.001
	DAY_PREC_1–12_	1	2.8e-02	3.2e-03	8.75	< 0.001
	DIST_RICE	0.13	-7.8e-05	1.6e-05	4.78	< 0.001
**Early + Late**	Intercept		6.96	0.53	12.97	< 0.001
	LST_21–32_	1	-0.15	0.021	7.24	< 0.001
	DAY_PREC_1–12_	1	1.7e-02	3.1e-03	5.04	< 0.001
	*NDWI*_*22–33*_	*0.6*	*-0.886*	*1.150*	*0.77*	*0.441*

The average weight and significance of variables remaining in the two best 'Early predictors only' and two best 'Early + Late predictors' models. Note that terms in italics are significant in some of the selected best models but not in others, and that overall, weighted model averaging procedures suggest that they are not significant.

## Discussion

The transmission of WNV is strongly linked to the abundance of the *Culex* mosquito vector [7,8], and many studies have focused on describing and quantifying habitat associations and spatio-temporal distributions of the vector species to guide implementation of effective control strategies [9,55]. In particular, early predictions of both the timing and intensity of future mosquito abundance will help to enable decision makers to apply effective prevention and control plans [10].

The current study aimed to identify early warning predictors of *Cx. pipiens* abundance and phenology, with the ultimate goal of improving entomological surveillance and focussing interventions to enable early detection of virus circulation in mosquitoes. To achieve this, we modelled the association between annual measures of mosquito abundance and phenology (start of the season and season length) and a set of environmental predictors.

Environmental predictors were selected based on published evidence of their importance to mosquito populations, and were averaged across twelve week periods in order to test the effect of variation at a seasonal scale, rather than focusing on daily or weekly fluctuations (e.g. [32]).

Our results indicate that warm temperatures during the early period (prior to the main mosquito season) lead to an earlier start, and extend the duration of the mosquito season (SEASL), but are not associated with a significant increase in abundance. This is likely to result from the acceleration of mosquito development rates driven by higher temperatures [20]. Higher temperatures during the late period (encompassing the main period of mosquito host seeking activity) are similarly associated with increased season length, but also with a decrease in total abundance. This latter result is opposite to the one found by [32] but is coherent with the observed captures: for instance 2003 was the hottest summer during the current study, and also the year with the least captures. This is also consistent with results obtained from laboratory experiments where adult survival and longevity of *Cx. pipiens* were negatively affected by high temperatures [56]. In addition, when high temperatures during summer are associated with low precipitation, as was the case in 2003, the combined effects of very hot and dry conditions are likely to cause rapid drying of aquatic breeding sites, with a consequent negative impact on mosquito populations. Recent observations in north-eastern Italy corroborate the negative impact of high summer temperatures, revealing a significant decline in populations when temperatures approached the maximum tolerance for *Cx. pipiens* over a prolonged period [57].

Early period precipitation postponed and shortened the activity of host-seeking mosquitoes, but at the same time was associated with greater abundance. Conversely, precipitation during the late period was associated with an extension of the season. An association between increased abundance and early period precipitation is probably associated with the increase in formation and persistence of mosquito breeding sites while more days of precipitation during the late period would prolong the existence of breeding pools, thus sustaining mosquito populations later in the year [19].

Higher values for environmental water (NDWI) during the early period were associated with an earlier start to the season and an increase in season length. These results highlight the importance of suitable breeding habitat, including surface water as well as vegetation water content [26,43,44]. Good levels of moisture, especially in the soil, are a fundamental requirement for the formation and persistence of mosquito breeding sites [43].

Although the two physical distances (to rice fields, and to urban areas) do not seem to be very important for *Cx. pipiens* in the current study, the negative association between abundance and distance from rice fields suggests that this land use provides important habitat in north-western Italy. This result was confirmed by larval collection of *Cx. pipiens* in rice-fields. Distances to urban areas were never selected in any of our models, suggesting that in this region of Italy urban settlements are not an important breeding habitat for *Cx. pipiens*, although it is possible that habitat type causes a bias in trap attractiveness. This is different to a number of other studies, carried out in the United States and Europe, where it has been shown that *Cx. pipiens* prefers urban settlements [12,25,36]. These preferences in the US may reflect differences in the ecology of *Cx. pipiens* in the Old, versus the New World, or may reflect differences in the biogeography of the two regions. Alternatively, such differences may reflect the presence of different forms of the species. Form *pipiens* prefers a more rural habitat, while *molestus* is more urban [58]. The form present in the eastern Piedmont area has not been definitively identified, but the relatively infrequent bites to humans (pers. obs) makes *pipiens* (which are predominantly bird-feeding) the more likely. Although [32] present spatial analyses (based on the same area as the current study) in which the highest abundances of *Cx. pipiens* were close to urban areas, the term was not significant in their final model. The equivocal nature of the results suggested by [32], and the lack of support for urban preference in the current study, using a longer time-series, supports a view that urban areas are of limited importance to *Cx. pipiens* in north western Italy.

## Conclusions

Although a wide range of environmental and non-environmental factors are involved in West Nile Virus outbreaks [5], the current study indicates that basic climatic monitoring data collected early in the year, in conjunction with local land use, can be used to provide early warning vector population dynamics, and therefore potential transmission risk. Overall, our analysis suggests that the early period of the year (prior to the start of the mosquito season) is very important to *Cx. pipiens* population dynamics: improvements to model accuracy by inclusion of the late period (during the main period of host seeking activity) were minimal. This result is particularly important in view of the need for timely implementation of mosquito control actions. The models developed are suitable for application in other areas where climate and land use are similar, while the principles used in model design can be applied across any area where mosquito population data and environmental data can be obtained. This has implications not only for West Nile Virus, but also for a wide range of other diseases that could be limited by mosquito control.

## Competing interests

The authors declare that they have no competing interests.

## Authors’ contributions

LBolzoni, GM, RR and AR conceived of the study, and participated in its design and coordination. LD, MM and MN collated spatial data in GRASS GIS. LBolzoni, GM and RR performed statistical analyses. LBalbo, LBertolotti, MG and AM coordinated the mosquito collections and built the original dataset. EC, GM and RR drafted the manuscript. AR contributed to interpretation and critical review. All authors participated in the revision of the manuscript and approved the submitted version.

## Supplementary Material

Additional file 1 **Section A.** Aggregation of environmental data over a range of time windows. Table A1. Significance of coefficients. Table A2. Number of changes of coefficient sign. Table A3. Minimum values of model AIC. Table A4. Average values of model AIC. **Section B**. Selection of the optimum 12 week time window using variation in AIC. Figure B1. Variation in AIC of preliminary models using 12 week aggregation period. **Section C**. Model selection tables. Table C1. The ten best full models predicting start of the mosquito season. Table C2. The ten best full models predicting length of the mosquito season using early period data only. Table C3. The ten best full models predicting season length using early and late period data. Table C4. The ten best full models predicting mosquito abundance using early period data only. Table C5. The ten best full models predicting mosquito abundance using early and late period data. Click here for file
