# Supplementary material for: Early warning of West Nile virus mosquito vector: climate and land use models successfully explain phenology and abundance of Culex pipiens mosquitoes in north-western Italy
Source: Parasit Vectors. 2014 Jun 12;7:269. doi: 10.1186/1756-3305-7-269 (PMC4061321; doi:10.1186/1756-3305-7-269)
Supplement: Additional file 1 — Section A. Aggregation of environmental data over a range of time windows. Table A1. Significance of coefficients. Table A2. Number of changes of coefficient sign. Table A3. Minimum values of model AIC. Table A4. Average values of model AIC. Section B. Selection of the optimum 12 week time window using variation in AIC. Figure B1. Variation in AIC of preliminary models using 12 week aggregation period. Section C. Model selection tables. Table C1. The ten best full models predicting start of the mosquito season. Table C2. The ten best full models predicting length of the mosquito season using early period data only. Table C3. The ten best full models predicting season length using early and late period data. Table C4. The ten best full models predicting mosquito abundance using early period data only. Table C5. The ten best full models predicting mosquito abundance using early and late period data. [file 1756-3305-7-269-S1.docx]

**A. Aggregation of environmental data over a range of time windows: preliminary analyses**

In order to select an appropriate period of time for aggregation of environmental data, we ran preliminary analyses comparing single model predictions of mosquito indices (ON, SEASL and TOTAL). Explanatory variables were the environmental predictors (DAY_PREC, LST or NDWI), and data for each environmental predictor were summed (DAY_PREC) or averaged (LST and NDWI) within each temporal window, using a range of aggregation periods: 1, 2, 4, 8, 12 weeks.

Comparisons were therefore made between:

- 1 week aggregation, producing 33 temporal windows from week 1 until week 33;

- 2 week aggregation, producing 32 temporal windows from weeks 1-2 until weeks 32-33;

- 4 week aggregation, producing 30 temporal windows from weeks 1-4 until weeks 30-33;

- 8 week aggregation, producing 26 temporal windows from weeks 1-8 until weeks 26-33;

- 12 week aggregation, producing 22 temporal windows from weeks 1-12 until weeks 22-33.

To make comparisons between models we looked at:

- The % of models with significant coefficients (Table A1).

- Consistency – estimated by how many times the coefficients from models using two consecutive temporal windows changed their sign (Table A2).

- Minimum and Mean values of model AIC (Tables A3 and A4)

|  |  |  |  |  |  |  |
| --- | --- | --- | --- | --- | --- | --- |

Table A1: Significance of coefficients (%).

|  |  | **Aggregation period (weeks)** | | | | |
| --- | --- | --- | --- | --- | --- | --- |
|  |  | 1 | 2 | 4 | 8 | 12 |
| ON | DAY_PREC | 0.95 | 0.75 | 0.78 | 0.93 | 1.00 |
|  | LST | 0.67 | 0.70 | 0.94 | 1.00 | 1.00 |
|  | NDWI | 0.38 | 0.42 | 0.40 | 0.83 | 1.00 |
| SEASL | DAY_PREC | 0.70 | 0.63 | 0.77 | 0.77 | 0.86 |
|  | LST | 0.61 | 0.59 | 0.77 | 0.88 | 1.00 |
|  | NDWI | 0.20 | 0.29 | 0.18 | 0.28 | 0.29 |
| TOTAL | DAY_PREC | 0.58 | 0.63 | 0.63 | 0.81 | 0.95 |
|  | LST | 0.70 | 0.75 | 0.73 | 0.65 | 0.64 |
|  | NDWI | 0.20 | 0.25 | 0.27 | 0.28 | 0.07 |
| All models | | 0.55 | 0.56 | 0.61 | 0.71 | 0.76 |

Table A2: Number of changes of coefficient sign.

|  |  | Aggregation period (weeks) | | | | |
| --- | --- | --- | --- | --- | --- | --- |
|  |  | 1 | 2 | 4 | 8 | 12 |
| ON | DAY_PREC | 7 | 5 | 3 | 0 | 0 |
|  | LST | 3 | 3 | 0 | 0 | 0 |
|  | NDWI | 0 | 2 | 0 | 0 | 0 |
| SEASL | DAY_PREC | 12 | 8 | 4 | 2 | 1 |
|  | LST | 9 | 7 | 2 | 0 | 0 |
|  | NDWI | 4 | 4 | 2 | 2 | 2 |
| TOTAL | DAY_PREC | 16 | 6 | 4 | 0 | 0 |
|  | LST | 6 | 6 | 4 | 2 | 2 |
|  | NDWI | 6 | 2 | 1 | 1 | 1 |
| All models | | 63 | 43 | 20 | 7 | 6 |
|  |  |  |  |  |  |  |

Table A3: Minimum values of model AIC.

|  |  | Aggregation period (weeks) | | | | |
| --- | --- | --- | --- | --- | --- | --- |
|  |  | 1 | 2 | 4 | 8 | 12 |
| ON | DAY_PREC | 4860.36 | 4729.09 | 4733.10 | 4726.25 | 4733.78 |
|  | LST | 4862.16 | 4744.36 | 4750.51 | 4748.47 | 4730.55 |
|  | NDWI | 4438.35 | 4781.76 | 4786.31 | 4796.04 | 4801.20 |
| SEASL | DAY_PREC | 4865.77 | 4731.22 | 4734.31 | 4731.77 | 4724.15 |
|  | LST | 4866.80 | 4749.80 | 4751.50 | 4751.80 | 4719.20 |
|  | NDWI | 4438.79 | 4779.01 | 4783.42 | 4792.01 | 4780.82 |
| TOTAL | DAY_PREC | 763.36 | 618.80 | 634.67 | 637.49 | 651.26 |
|  | LST | 764.49 | 656.40 | 624.28 | 610.83 | 623.34 |
|  | NDWI | 688.14 | 700.33 | 700.12 | 695.43 | 702.88 |
| All models | | 3394.25 | 3387.86 | 3388.69 | 3387.79 | 3385.24 |

Table A4: Average values of model AIC.

|  |  | Aggregation period (weeks) | | | | |
| --- | --- | --- | --- | --- | --- | --- |
|  |  | 1 | 2 | 4 | 8 | 12 |
| ON | DAY_PREC | 4912.25 | 4786.94 | 4778.19 | 4759.43 | 4759.40 |
|  | LST | 4909.12 | 4785.25 | 4781.99 | 4771.03 | 4767.06 |
|  | NDWI | 4777.65 | 4793.08 | 4797.79 | 4799.93 | 4801.62 |
| SEASL | DAY_PREC | 4912.70 | 4788.84 | 4786.97 | 4780.72 | 4774.71 |
|  | LST | 4927.64 | 4807.04 | 4811.70 | 4812.60 | 4797.89 |
|  | NDWI | 4777.19 | 4793.76 | 4798.80 | 4801.72 | 4792.06 |
| TOTAL | DAY_PREC | 809.38 | 699.33 | 703.82 | 694.19 | 697.41 |
|  | LST | 804.86 | 691.83 | 692.36 | 678.22 | 682.99 |
|  | NDWI | 805.29 | 712.60 | 716.84 | 698.18 | 707.00 |
| All models | | 3515.12 | 3428.74 | 3429.83 | 3421.78 | 3420.02 |

The 12-week aggregation performed better than all other choices, across all of the given parameters. The 12 week period gave on average the highest percentage of significant coefficients (76%), the lowest number of changes of coefficient sign (6), and the lowest values of MIN and MEAN AIC (3385.24 and 3420.02 respectively).

**B. Selection of the optimum 12 week time window using variation in AIC (ΔAIC)**

The time window producing the lowest AIC was selected for inclusion in full models.

|  | **DAY PREC** | **LST** | **NDWI** |
| --- | --- | --- | --- |
| **ON** | **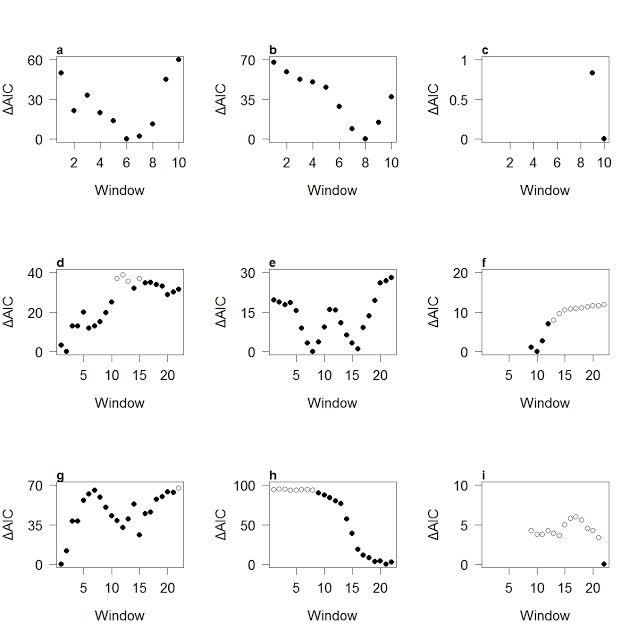** | | |
| **SEASL** |  |  |  |
| **TOTAL** |  |  |  |

**Figure B1**. Variation in AIC (ΔAIC) of preliminary models using 12 week aggregation period. Comparisons are made between different temporal windows for DAY PREC (left column), LST (central column) and NDWI (right column) as predictors of the start of mosquito season ON (upper row), season length SEASL (central row) and mosquito abundance TOTAL (lower row). Temporal windows are labelled according to the starting week, i.e. 1 (weeks 1-12), 2 (weeks 2-13), etc., to 21 (weeks 21-33). Filled dots indicate significant coefficients (p<0.05).

**C. Model selection tables**

Table C1: The ten ‘best’ full models predicting start of the mosquito season (ON) – those with lowest AIC values obtained from model selection. For each model we report AIC, the difference in AIC with respect to the best model (ΔAIC), the Akaike weight (wAIC) and R^2^.

| **Models** | **AIC** | **ΔAIC** | **wAIC** | **R^2^** |
| --- | --- | --- | --- | --- |
| ON ~ LST_8.19_ + DAY_PREC_6.17_ + NDWI_10.21_ | 4687.93 | 0.00 | 0.962207 | 0.258 |
| ON ~ DIST_URBAN + LST_8.19_ + DAY_PREC_6.17_ + NDWI_10.21_ | 4695.29 | 7.36 | 0.024327 | 0.266 |
| ON ~ LST_8.19_ + NDWI_10.21_ | 4697.15 | 9.22 | 9.59E-03 | 0.240 |
| ON ~ DAY_PREC_6.17_ + NDWI_10.21_ | 4700.45 | 12.52 | 1.84E-03 | 0.241 |
| ON ~ DIST_RICE + LST_8.19_ + DAY_PREC_6.17_ + NDWI_10.21_ | 4701.11 | 13.17 | 1.33E-03 | 0.258 |
| ON ~ LST_8.19_ + DAY_PREC_6.17_ | 4703.46 | 15.52 | 4.10E-04 | 0.247 |
| ON ~ DIST_URBAN + DAY_PREC_6.17_ + NDWI_10.21_ | 4706.06 | 18.12 | 1.12E-04 | 0.252 |
| ON ~ DIST_URBAN + LST_8.19_ + NDWI_10.21_ | 4706.28 | 18.35 | 9.99E-05 | 0.245 |
| ON ~ DIST_RICE + LST_8.19_ + NDWI_10.21_ | 4708.90 | 20.97 | 2.69E-05 | 0.243 |
| ON ~ DIST_URBAN + LST_8.19_ + DAY_PREC_6.17_ | 4710.08 | 22.14 | 1.49E-05 | 0.257 |

Table C2: The ten ‘best’ full models predicting length of the mosquito season (SEASL) using early period data only – those with lowest AIC values obtained from model selection. For each model we report AIC, the difference in AIC with respect to the best model (ΔAIC), the Akaike weight (wAIC) and R^2^.

| **Models** | **AIC** | **ΔAIC** | **wAIC** | **R^2^** |
| --- | --- | --- | --- | --- |
| SEASL ~ LST_8.19_ + DAY_PREC_2.13_ + NDWI_10.21_ | 3380.28 | 0.00 | 0.767278 | 0.141 |
| SEASL ~ DAY_PREC_2.13_ + NDWI_10.21_ | 3382.85 | 2.57 | 0.212613 | 0.135 |
| SEASL ~ LST_8.19_ + NDWI_10.21_ | 3388.97 | 8.69 | 9.95E-03 | 0.114 |
| SEASL ~ DIST_URBAN + LST_8.19_ + DAY_PREC_2.13_ + NDWI_10.21_ | 3389.89 | 9.61 | 6.28E-03 | 0.153 |
| SEASL ~ DIST_URBAN + DAY_PREC_2.13_ + NDWI_10.21_ | 3391.68 | 11.39 | 2.57E-03 | 0.148 |
| SEASL ~ LST_8.19_ + DAY_PREC_2.13_ | 3394.00 | 13.72 | 8.05E-04 | 0.127 |
| SEASL ~ DIST_RICE + LST_8.19_ + DAY_PREC_2.13_ + NDWI_10.21_ | 3396.14 | 15.86 | 2.76E-04 | 0.143 |
| SEASL ~ DAY_PREC_2.13_ | 3398.28 | 17.99 | 9.49E-05 | 0.116 |
| SEASL ~ DIST_RICE + DAY_PREC_2.13_ + NDWI_10.21_ | 3399.48 | 19.20 | 5.2E-05 | 0.135 |
| SEASL ~ DIST_URBAN + LST_8.19_ + NDWI_10.21_ | 3400.02 | 19.73 | 3.98E-05 | 0.123 |

Table C3: of the ten ‘best’ full models predicting season length (SEASL) using early and late period data – those with lowest AIC values obtained from model selection. For each model we report AIC, the difference in AIC with respect to the best model (ΔAIC), the Akaike weight (wAIC) and R^2^.

| **Models** | **AIC** | **ΔAIC** | **wAIC** | **R^2^** |
| --- | --- | --- | --- | --- |
| SEASL ~ NDWI_10.21_ + LST_16.27_ + DAY_PREC_20.31_ | 3374.32 | 0.00 | 0.283167 | 0.156 |
| SEASL ~ LST_8.19_ + NDWI_10.21_ + LST_16.27_ + DAY_PREC_20.31_ | 3374.41 | 0.09 | 0.27071 | 0.156 |
| SEASL ~ LST_8.19_ + DAY_PREC_2.13_ + NDWI_10.21_ + LST_16.27_ + DAY_PREC_20.31_ | 3376.08 | 1.76 | 0.117533 | 0.16 |
| SEASL ~ DAY_PREC_2.13_ + NDWI_10.21_ + LST_16.27_ + DAY_PREC_20.31_ | 3376.08 | 1.76 | 0.117364 | 0.16 |
| SEASL ~ LST_8.19_ + DAY_PREC_2.13_ + NDWI_10.21_ + LST_16.27_ | 3376.58 | 2.26 | 0.091288 | 0.15 |
| SEASL ~ DAY_PREC_2.13_ + NDWI_10.21_ + LST_16.27_ | 3377.55 | 3.23 | 0.056259 | 0.147 |
| SEASL ~ LST_8.19_ + NDWI_10.21_ + LST_16.27_ | 3378.36 | 4.04 | 0.03753 | 0.138 |
| SEASL ~ LST_8.19_ + DAY_PREC_2.13_ + NDWI_10.21_ | 3380.28 | 5.96 | 0.014372 | 0.141 |
| SEASL ~ DAY_PREC_2.13_ + NDWI_10.21_ | 3382.85 | 8.53 | 3.98E-03 | 0.135 |
| SEASL ~ NDWI_10.21_ + LST_16.27_ | 3383.76 | 9.44 | 2.52E-03 | 0.125 |

Table C4: The ten ‘best’ full models predicting mosquito abundance (TOTAL) using early period data only – those with lowest AIC values obtained from model selection. For each model we report AIC, the difference in AIC with respect to the best model (ΔAIC), the Akaike weight (wAIC) and R^2^.

| **Models** | **AIC** | **ΔAIC** | **wAIC** | **R^2^** |
| --- | --- | --- | --- | --- |
| TOTAL ~ DAY_PREC_1.12_ | 634.19 | 0.00 | 0.79395 | 0.464 |
| TOTAL ~ DIST_RICE + DAY_PREC_1.12_ | 637.92 | 3.73 | 0.12304 | 0.488 |
| TOTAL ~ LST_10.21_ + DAY_PREC_1.12_ | 638.90 | 4.71 | 0.075289 | 0.467 |
| TOTAL ~ DIST_RICE + LST_10.21_ + DAY_PREC_1.12_ | 644.04 | 9.85 | 5.76E-03 | 0.489 |
| TOTAL ~ DIST_URBAN + DAY_PREC_1.12_ | 647.14 | 12.95 | 1.22E-03 | 0.473 |
| TOTAL ~ DIST_URBAN + DIST_RICE + DAY_PREC_1.12_ | 648.49 | 14.30 | 6.22E-04 | 0.501 |
| TOTAL ~ DIST_URBAN + LST_10.21_ + DAY_PREC_1.12_ | 652.21 | 18.03 | 9.67E-05 | 0.476 |
| TOTAL ~ DIST_URBAN + DIST_RICE + LST_10.21_ + DAY_PREC_1.12_ | 655.10 | 20.92 | 2.28E-05 | 0.501 |
| TOTAL ~ 1 | 692.45 | 58.27 | 1.77E-13 | 0.365 |
| TOTAL ~ LST_10.21_ | 693.21 | 59.03 | 1.21E-13 | 0.375 |

Table C5: The ten ‘best’ full models predicting mosquito abundance (TOTAL) using early and late period data – those with lowest AIC values obtained from model selection. For each model we report AIC, the difference in AIC with respect to the best model (ΔAIC), the Akaike weight (wAIC) and R^2^.

| **Models** | **AIC** | **ΔAIC** | **wAIC** | **R^2^** |
| --- | --- | --- | --- | --- |
| TOTAL ~ DAY_PREC_1.12_ + LST_21.32_ + NDWI_22.33_ | 592.74 | 0.00 | 0.494334 | 0.524 |
| TOTAL ~ DAY_PREC_1.12_ + LST_21.32_ | 593.42 | 0.68 | 0.351739 | 0.523 |
| TOTAL ~ DIST_RICE + DAY_PREC_1.12_ + LST_21.32_ + NDWI_22.33_ | 597.94 | 5.20 | 0.036789 | 0.543 |
| TOTAL ~ LST_10.21_ + DAY_PREC_1.12_ + LST_21.32_ + NDWI_22.33_ | 598.29 | 5.55 | 0.030896 | 0.526 |
| TOTAL ~ DAY_PREC_1.12_ + LST_21.32_ + DAY_PREC_15.26_ + NDWI_22.33_ | 598.88 | 6.14 | 0.022952 | 0.530 |
| TOTAL ~ LST_10.21_ + DAY_PREC_1.12_ + LST_21.32_ | 599.18 | 6.44 | 0.019778 | 0.525 |
| TOTAL ~ DAY_PREC_1.12_ + LST_21.32_ + DAY_PREC_15.26_ | 599.46 | 6.72 | 0.017162 | 0.529 |
| TOTAL ~ DIST_RICE + DAY_PREC_1.12_ + LST_21.32_ | 602.01 | 9.27 | 4.80E-03 | 0.539 |
| TOTAL ~ DIST_URBAN + DAY_PREC_1.12_ + LST_21.32_ + NDWI_22.33_ | 602.10 | 9.36 | 4.58E-03 | 0.537 |
| TOTAL ~ DIST_URBAN + DAY_PREC_1.12_ + LST_21.32_ | 602.48 | 9.73 | 3.80E-03 | 0.536 |
